# Supplementary material for: Prevalence of Adverse Childhood Experiences in the First Decade of Life: A Study in the Portuguese Cohort, Generation XXI
Source: Int J Environ Res Public Health. 2022 Jul 8;19(14):8344. doi: 10.3390/ijerph19148344 (PMC9324541; doi:10.3390/ijerph19148344)
Supplement: Supplementary file 1 [file ijerph-19-08344-s001.zip › ijerph-1755827-supplementary.pdf]

Supplementary Table S1. Descriptive characteristics of the 5,295 10-year children from the Generation XXI enrolled in the study.

	**n (%)**
Boys	2697 (50.9)
Age (mean, ±SD)	10.15 (0.34)
ACEs (yes)	5092 (96.2)
Dimensions of ACEs (cumulative)	
0 dimensions	203 (3.8)
1 dimension	671 (12.7)
2 dimensions	1308 (24.7)
3 dimensions	1565 (29.5)
4 dimensions	1174 (22.2)
5 dimensions	374 (7.1)
Living in a one-parent family or other structure	1149 (21.7)
Household income (≤1,000€)	1371 (26.9)
Parents' educational level (≤9 years)	1220 (29.4)
Parents' unemployment (one or both)	1052 (40.3)
Medical diagnosis of any disease	335 (6.4)
Medical diagnosis of asthma	420 (8.0)
Obesity	872 (16.5)
Low consumption of fruits and vegetables	1776 (33.9)
Excess of screen activities (>480 minutes/week)	913 (17.4)
